# Determinants of Default from Pulmonary Tuberculosis Treatment in Kuwait

**DOI:** 10.1155/2014/672825

**Published:** 2014-04-06

**Authors:** Qing Zhang, Mohamed Gaafer, Ibrahim El Bayoumy

**Affiliations:** ^1^Tuberculosis Diagnosis and Treatment Center, Shanghai, Pulmonary Hospital, School of Medicine, Tongji University, 507 Zheng Min Road, Shanghai 200433, China; ^2^Community, Environmental and Occupational Medicine Department, Minufiya University, Egypt; ^3^Public Health Department, Tanta Faculty of Medicine, Egypt; ^4^Ports and Borders Health Division, P.O. Box 35180, 36052 Shaab, Kuwait

## Abstract

*Objectives.* To determine the prevalence and risk factors of default from pulmonary tuberculosis treatment in Kuwait. *Design.* Retrospective study. Patients and methods. We studied all patients who were registered for pulmonary tuberculosis treatment between January 1, 2010, and December 31, 2012, and admitted into TB wards in El Rashid Center or treated in the outpatient clinic in TB Control Unit. *Results.* There were 110 (11.5%) patients who defaulted from treatment. Fifty-six percent of those who defaulted did so in the first 2 months of treatment and 86.4% of them were still bacteriologically positive at the time of default. Key risk factors associated with noncompliance were male sex, low educational level, non-Kuwaiti nations, history of default, and history of concomitant diabetes mellitus, liver disease, or lung cancer. Multiple drug resistance was also associated with default from treatment. *Conclusion.* Default from treatment may be partially responsible for the persistent relatively high rates of tuberculosis in Kuwait. Health professionals and policy makers should ensure that all barriers to treatment are removed and that incentives are used to encourage treatment compliance.

## 1. Introduction

Tuberculosis is a leading cause of morbidity and mortality worldwide; 2 billion people, equal to one-third of the world's total population, are infected with TB bacilli, the microbes that cause TB [1].

In Kuwait, the incidence of tuberculosis was very high in late 1960s, at nearly 270 cases per 100 000 population. The National Tuberculosis Programme (NTP) has been working hard to bring tuberculosis under control. The NTP adopted the Directly Observed Treatment Short course (DOTS) strategy in 1998, expanded it rapidly, and achieved 100% DOTS population coverage. With the sustained efforts of the NTP and the progressive improvement in the socioeconomic situation, the notification rate of all forms and smear positive tuberculosis reached 21.1 and 9.0 per 100,000 population in 2006 (resp.). This means that 645 people developed tuberculosis last year and 274 of them were infectious (i.e., smear positive) [2].

Early diagnosis of tuberculosis and effective treatment are the key elements in reduction of transmission of infection and finally achieving elimination of TB [3]. World Health Organization (WHO) had set the international target value for a favorable treatment outcome at 85% [4]. In many industrialized countries with good treatment facilities and a secured supply of drugs free of charge for patients, treatment results have not reached the targets set by WHO yet [5, 6].

Treatment outcome monitoring is a core part of surveillance necessary to succeed in tuberculosis elimination [7]. The WHO has published a recommendation for assessing the outcome of tuberculosis treatment in the 1990s [8], revised recently [9, 10].

The specific reasons for unsuccessful outcomes are important in order to improve treatment systems. In a recent outcome analysis from Norway, where the large majority of TB cases are in immigrants, only high age and isoniazid (INH) resistance were significant risk factors for unsuccessful outcome [11]. In earlier studies, high age, alcoholism, HIV infection, male sex, and immigration have been associated with unfavorable outcomes [12].

The currently recommended minimum duration of treatment is 6 months, which, although much shorter than the previously recommended 12 to 24 months, is still very long. According to WHO, DOTS ensures successful treatment of patients with tuberculosis [13]. There are still patients who are not compliant to DOTS and default from treatment.

Poor case management, often because of nonadherence to treatment, has emerged as the most important factor in the resurgence of tuberculosis and the appearance of multiple drug resistance, MDR (resistance to two or more of the primary drugs, isoniazid and rifampin), and extensively Drug Resistance, XDR (resistance to at least isoniazied and rifampin among the first-line anit-TB drugs and among second-line drugs, is resistant to any fluoroquinolone and at least one of three injectable drugs) [14]. The prolonged duration of treatment, the need for multiple drugs, and socioeconomic factors are the main reasons for nonadherence to treatment.

This study aims to assess the determinants and predictors of default from pulmonary tuberculosis treatment in Kuwait.

## 2. Patients and Methods

The State of Kuwait is located in the Gulf area and has a population of approximately 3.3 million, 68% of whom are expatriates seeking residency for work [15]. 60% of residents come from South East Asia where tuberculosis is endemic and constitutes 40–45% of worldwide TB cases [16].

We studied all patients who were registered for pulmonary tuberculosis treatment between January 1, 2010 and December 31, 2012 and admitted in TB wards in* El Rashid *Center or treated in theoutpatient clinic in TB Control Unit. These governmental facilities concentrate the treatment of all pulmonary TB patients around the country.

Default was defined as failure to collect medication for more than 2 consecutive months after the date of the last attendance. To examine the determinants and risk factors of default from treatment, we used a nested case control study design. For each case of default* (110 cases)*, three controls were randomly selected* (330 controls)* from cases that cured or completed treatment, using computer-generated random numbers.

Data of patients treated were obtained from programme forms (treatment cards) that were submitted by chest physicians at the onset of pulmonary tuberculosis treatment and thereafter. The forms had the following information: patient name, starting date of treatment, age, sex, history of treatment, the extent of disease, and the case category (new, relapse, treatment after default, and treatment after failure). The forms also listed the type of drug regimen used, the frequency of drug treatment, side effects, bacteriological status before treatment and at 2 and 5 or 6 months from the start of treatment, drug sensitivity of microorganisms, and the treatment outcome. In addition, we reviewed medical records at TB Control Unit for patients with missing forms, missing information, and inconsistent information between forms. The definitions used in this study were modified from those according to the International Union Against Tuberculosis and Lung Diseases (IUATLD) [17].

### 2.1. Statistical Analysis

The data were analyzed using the Statistical Package for the Social Sciences (Windows version 12; SPSS Inc., Chicago, US). The differences in clinical features between the cases of default and the controls were tested by Chi squared analysis. Multiple logistic regression was used to analyze the association between various risk factors and treatment default. This study was approved by the Ethics Committee of NTP.

## 3. Results

See Tables 1, 2, 3, 4, and 5.

## 4. Discussion

In Kuwait, the policy of National Tuberculosis Program (NTP) is to manage all cases of pulmonary tuberculosis with DOTS strategy in two governmental facilities, outpatient clinic in TB Control Unit, and TB wards in El Rashid Center. The cost of drug treatment is not an issue in Kuwait, because all antituberculous drugs are free of charge for both Kuwaiti and non-Kuwaiti. The infrastructure for DOTS in Kuwait is very good.

Default from treatment is unlikely to be solely responsible for the current stagnant rate of tuberculosis at a relatively high level. One hundred and ten (11.5%) of patients who registered for treatment of pulmonary tuberculosis between January 1, 2010 and December 31, 2012 defaulted from treatment. 86.4% of them had positive results to smear or culture tests, indicating that they were still potentially infectious. However, the treatment Cure Rate (with exclusion of who left the country) was about 88.6%, which reaches the target set by the WHO for treatment completion [1].

According to published studies, one smear and culture positive patient with pulmonary tuberculosis infects, on average, about twelve others each year, while each culture positive patient infects two others [18]. Hence, the patients who defaulted from treatment in study period might have infected 780 individuals in the first year after default from treatment. Furthermore, about 10% to 15% of infected individuals develop the disease after infection and half of them may be infectious and in turn can transmit the disease to others [19]. So, these defaulters might have added substantially to the pool of infectious people.

Because of other factors, such as death, natural remission, retreatment of some cases, and the effect of partial treatment on the viability of the bacilli, it is impossible to detect exactly the impact of default from treatment to the overall rate of tuberculosis in Kuwait. But the mean duration of infectivity of each case of tuberculosis is estimated to be 2 years [17], so the additional annual risk of infection attributable to patients who defaulted from treatment in Kuwait is about 0.02%.

In this study, we found that a history of default from treatment is the strongest predictor of non-compliance, followed by the presence of concomitant diabetes mellitus, liver disease, lung cancer, low educational level, male sex, and non-Kuwaiti nationalities. Defaulting was more frequently encountered among non-Kuwaiti than Kuwaiti (79.1% and 20.9%, resp.), especially those coming from south East Asia, where TB is endemic and constitutes 40–45% of worldwide TB cases [20]: Indian (40.9%), Bangladeshi (20.9%), Indonesian (13.6%), Pakistani (10%), Philippine (6.4%), and other nationalities (4.6%).

Most published studies had shown that HIV infection, homelessness, smoking, alcohol and drug misuse, psychiatric illness, and poverty are all risk factors for noncompliance to treatment [21]. In our study, these data were not available in the programme forms.

The risk of MDR tuberculosis was significantly higher among defaulters than non-defaulters, which is consistent with most of the published results [22]. This finding is especially worrisome, because all patients were still bacteriologically positive at the time of treatment default.

Legal sanction against defaulters has been attempted in the United States in the mid-1980s, when there was a dramatic rise in the rate of tuberculosis and MDR tuberculosis among HIV-infected individuals [23]. Although there was widespread support for such legal action, there were concerns that these powers might be abused for use as a means of social control, and many believed that it was unfair to detain patients when their ability to comply with treatment was affected by the lack of housing, primary health care, and services for substance users [24].

In the United Kingdom, legislation allows for the detention of an individual with a notifiable disease that is a threat to others, but this legislation is rarely used [25]. The moral issues in the use of coercion and detainment in dealing with nonadherence in the diagnosis and treatment of tuberculosis have been widely debated [26, 27]. Although coercion and detainment have been found by ethicists to be a morally acceptable strategy to fight the spread of tuberculosis, their use to support strategies that improve treatment compliance must be sensitive to national and cultural differences and not simply be based on perceived successes elsewhere.

Due regard must be paid to the possibility that such negative measures may aggravate the social stigma and discrimination which still surround this important airborne disease. Even if we can achieve 100% case detection, the strategy will still not work unless patients come forward for treatment.

On the other hand, it has been shown that successful DOT programmes with a high rate of treatment completion (86%–97%) are those that provide incentives such as shelter for the homeless, methadone and rehabilitation programmes for drug abusers, food coupons, and money for transportation; some programmes also provide educational opportunities, while others make use of occupation settings to administer the drugs [28, 29]. Directly observed therapy programmes without such incentives are less successful, with treatment completion rate of 85.0% to 87.5% [30].

The current DOT programme in Kuwait uses a combination of incentives and enablers, which include education for patients and their families, referrals for other social services, as well as use of outreach teams, and the tracing of defaulters. Greater efforts are required in the education of the patients and public to increase their understanding of the importance of treatment completion for patients with tuberculosis.

## Figures and Tables

**Table 1:** Outcome of pulmonary tuberculosis treatment by sputum smear status (for the years 2004–2006).

Treatment outcome	Smear positive	Smear negative	Total
Cured	318 (44.3)	—	318 (33.4)
Completed	178 (24.8)	180 (76.3)	358 (37.5)
Died	5 (0.7)	0 (0.0)	5 (0.5)
Failed	0 (0.0)	0 (0.0)	0 (0.0)
Defaulted	**59 (8.2)**	**51 (21.6)**	**110 (11.5)**
Left the country	158 (22.0)	5 (2.1)	163 (17.1)

Total registered	718 (100.0)	236 (100.0)	954 (100.0)

Out of the 954 patients who were treated for tuberculosis in the study period, 110 (11.5%) refused treatment or failed to attend the clinic for more than 2 consecutive months, thereby fulfilling the criteria for treatment default. One hundred fifty-eight smear positive TB cases (22.0% of smear positive) were new comer expatriates; they had taken antituberculous medications until sputum is converted, to be noninfectious during transportation, and then deported to their countries. Cure Rate (cured plus completed) for smear positive cases = 88.6% (with exclusion of who left the country) and 69.1% (without exclusion of who left the country).

**Table 2:** Demographic characteristics of defaulters and nondefaulters.

Variables	Defaulters (*n* = 110)	Nondefaulters (*n* = 330)	*P* value
Age group (years):			
0–19	4 (3.6)	15 (4.6)	0.18 (NS)
20–39	45 (40.9)	115 (34.8)	
40–59	39 (35.5)	100 (30.3)	
≥60	22 (20.0)	100 (30.3)	
Sex:			
Female	26 (23.6)	125 (37.8)	0.006
Male	84 (76.4)	200 (62.2)	
Nationality:			
Kuwaiti	4 (20.9)	50 (15.1)	0.002
Non-Kuwaiti	106 (79.1)	280 (84.9)	
Educational status:			
Undergraduate	88 (80.0)	185 (56.1)	<0.0001
Graduate and above	22 (20.0)	145 (43.9)	
Marital status:			
Single	71 (64.5)	187 (56.7)	0.12 (NS)
Married	20 (18.2)	98 (29.7)	
Divorced	11 (10.0)	28 (8.5)	
Widowed	8 (7.3)	17 (5.1)	
Case category:			
New case	90 (81.8)	290 (87.9)	0.15 (NS)
Treatment after default	14 (12.7)	6 (1.8)	<0.0001
Relapse	2 (1.8)	10 (3.0)	0.74 (NS)
Others	4 (3.6)	24 (7.3)	0.26 (NS)
Concomitant illness:	23 **(20.9)**	21 **(6.4)**	<0.0001
Diabetes mellitus	11 (10.0)	13 (3.9)	0.02
Liver disease as fibrosis	6 (5.4)	4 (1.2)	0.02
Lung cancer	4 (3.6)	1 (0.3)	0.02
Renal disease	2 (1.8)	3 (0.9)	0.79 (NS)

There was no significant difference in age or marital status between defaulters and nondefaulters. The defaulted group had more men than women, a higher proportion with low educational level (80.0%), and a history of default compared with the control group. The difference was statistically significant (*P* < 0.05).

Regarding nationality, defaulting was more frequently encountered among non-Kuwaiti than Kuwaiti (79.1% and 20.9%, resp.). Non Kuwaiti defaulters including Indian (40.9%), Bangladeshi (20.9%), Indonesian (13.6%), Pakistani (10%), Philippine (6.4%), and other nationalities (4.6%).

A higher proportion of cases than of controls had concomitant illnesses; the proportion with diabetes, liver diseases, and lung cancers were higher among those who defaulted from treatment (*P* < 0.05).

**Table 3:** Extent of disease, cavitation, bacteriology, and drug susceptibility pattern among defaulters and nondefaulters.

Variables	Defaulters (*n* = 110)	Nondefaulters (*n* = 330)	*P* value
Extent of disease:			
Minimal	66 (60.0)	210 (63.6)	0.96 (NS)
Moderate	32 (29.1)	85 (28.8)	
Advanced	12 (10.9)	29 (11.8)	
Cavitary disease:	22 (20.0)	65 (19.7)	0.83 (NS)
Bacteriological status:			
Smear and culture positive	43 (39.1)	128 (38.8)	0.92 (NS)
Smear positive only	16 (14.6)	57 (17.3)	
Culture positive only	36 (32.7)	102 (30.9)	
Smear and culture negative	15 (13.6)	43 (13.0)	
Drug susceptibility:			
Fully sensitive	97 (88.2)	295 (89.4)	0.79 (NS)
Resistant to one first-line drug	9 (8.2)	27 (8.2)	
Resistant to more than one first-line drug	4 (3.6)	8 (2.4)	
Multiple drug resistance:	4 (3.7)	1 (0.3)	0.01

There were no statistical significant differences between cases and controls regarding the extent of disease, presence of cavitation, or positive bacteriology (*P* > 0.05).

Whereas the proportion with resistance to one or more drugs was similar between cases and controls, the proportion with MDR was higher among cases (3.7) than among controls (0.3). Four patients with MDR who defaulted were still culture positive and one of them was also smear positive at the time of default.

**Table 4:** Distribution of defaulters by time of default and bacteriological status.

Start time of treatment	Smear and culture positive	Smear positive	Culture positive	Smear and culture negative	Total
No treatment	1 (2.3)	0 (0.0)	2 (5.6)	0 (0.0)	3 (2.7)
<2 weeks	3 (7.0)	4 (25.0)	3 (8.3)	2 (13.3)	12 (10.9)
2 weeks–2 months	16 (37.2)	9 (56.3)	19 (52.8)	3 (20.0)	47 (42.7)
2 weeks–4 months	10 (23.3)	2 (12.5)	8 (22.2)	3 (20.0)	23 (20.9)
>4 months	13 (30.2)	1 (6.2)	4 (11.1)	7 (46.7)	25 (22.8)

Total	43 (39.1)	16 (14.6)	36 (32.7)	15 (13.6)	110 (100.0)

Approximately 56% of patients who defaulted did so within the first 2 months of treatment. Of the 110 patients who defaulted, 95 (86.4%) were still bacteriologically positive (43 smear and culture positive, 16 smear positive, and 36 culture positive only) at the time of default.

**Table 5:** Odds ratios from multiple logistic regression analysis, examining the association between selected risk factors and treatment default.

Variables	Odds ratio	95% confidence interval	*P* value
Age (years)	0.95	0.96–1.1	0.56 (NS)
Sex:			
Male versus female	1.3	1.2–1.7	0.03
Educational status:			
Undergraduate versus graduate and above	1.9	1.6–4.3	0.01
Nationality:			
Non-Kuwaiti versus Kuwaiti	2.4	1.9–6.4	0.01
Cavitation:			
Yes versus no	0.9	0.7–1.5	0.81 (NS)
Case category:			
Relapse versus new case	0.8	0.5–1.4	0.74 (NS)
Previous default versus new case	1.6	1.1–2.9	0.01
Extent of disease:			
Moderate versus minimal	0.8	0.6–1.4	0.66 (NS)
Advanced versus minimal	0.6	0.5–1.2	0.74 (NS)
Concomitant illness:			
Diabetes: yes versus no	1.6	1.8–4.4	0.03
Liver disease: yes versus no	1.4	1.2–3.3	0.04
Lung cancer: yes versus no	1.3	1.1–2.7	0.04
Renal disease: yes versus no	0.9	0.6–1.2	0.35 (NS)
Drug susceptibility:			
Multiple drug resistance versus fully sensitive	2.1	1.3–5.6	0.01

Multiple logistic regression analyses were performed to determine the risk factors associated with default among those with pulmonary tuberculosis. The important risk factors associated with default included male sex (odds ratio [OR] = 1.3; 95% confidence interval [CI], 1.2–1.7), low educational level (OR = 1.9; 95% CI, 1.6–4.3), non-Kuwaiti nations (OR = 2.4; 95% CI, 1.9–6.4), a history of default (OR = 1.6; 95% CI, 1.1–2.9), diabetes mellitus (OR = 1.6; 95% CI, 1.8–4.4), liver disease (OR = 1.4; 95% CI, 1.2–3.3), and lung cancer (OR = 1.3; 95% CI, 1.1–2.7).
